# *Apol9a* regulates myogenic differentiation *via* the ERK1/2 pathway in C2C12 cells

**DOI:** 10.3389/fphar.2022.942061

**Published:** 2022-11-23

**Authors:** Xuan Jiang, Siyu Ji, Siyuan Cui, Rong Wang, Wei Wang, Yongquan Chen, Shenglong Zhu

**Affiliations:** ^1^ Wuxi School of Medicine, Jiangnan University, Wuxi, China; ^2^ School of Food Science and Technology, Jiangnan University, Wuxi, China; ^3^ The Wuxi No. 2 People’s Hospital, Wuxi, China; ^4^ Wuxi Translational Medicine Research Center and School of Translational Medicine, Jiangnan University, Wuxi, China

**Keywords:** obesity, myogenesis, Apol9a, ERK1/2, C2C12

## Abstract

**Background:** The rising prevalence of obesity and its complications is a big challenge for the global public health. Obesity is accompanied by biological dysfunction of skeletal muscle and the development of muscle atrophy. The deep knowledge of key molecular mechanisms underlying myogenic differentiation is crucial for discovering novel targets for the treatment of obesity and obesity-related muscle atrophy. However, no effective target is currently known for obesity-induced skeletal muscle atrophy.

**Methods:** Transcriptomic analyses were performed to identify genes associated with the regulation of myogenic differentiation and their potential mechanisms of action. C2C12 cells were used to assess the myogenic effect of *Apol9a* through immunocytochemistry, western blotting, quantitative polymerase chain reaction, RNA interference or overexpression, and lipidomics.

**Results:** RNA-seq of differentiated and undifferentiated C2C12 cells revealed that *Apol9a* expression significantly increased following myogenic differentiation and decreased during obesity-induced muscle atrophy. *Apol9a* silencing in these C2C12 cells suppressed the expression of myogenesis-related genes and reduced the accumulation of intracellular triglycerides. Furthermore, RNA-seq and western blot results suggest that *Apol9a* regulates myogenic differentiation through the activation of extracellular signal-regulated kinase 1/2 (ERK1/2). This assumption was subsequently confirmed by intervention with PD98059.

**Conclusion:** In this study, we found that *Apol9a* regulates myogenic differentiation *via* the ERK1/2 pathway. These results broaden the putative function of *Apol9a* during myogenic differentiation and provide a promising therapeutic target for intervention in obesity and obesity-induced muscle atrophy.

## Introduction

Obesity is widely reported to be a potential risk factor for type 2 diabetes mellitus (T2DM), a metabolic disorder typified by chronic hyperinsulinemia, hyperglycemia, and insulin resistance (Astrup and Finer, 2000; Lingvay et al., 2021). Diabetes is reportedly stimulated during obesity because of a failure in appropriate glucose utilization by skeletal muscle, which is the primary target site of insulin-stimulated glucose uptake (DeFronzo and Tripathy, 2009; Lingvay et al., 2021; Mengeste et al., 2021). Obesity-related ectopic fat deposition induces biological dysfunction in skeletal muscle, such as insulin resistance (IR), mitochondrial dysfunction, and inflammation (Wu and Ballantyne, 2017). These processes further exacerbate skeletal muscle loss and physical dysfunction. Maintaining skeletal muscle health is fundamental for general health.

Myogenesis, which includes satellite cell activation, myoblast differentiation, and myotube formation, is responsible for the maintenance of skeletal muscle mass and integrity in general (Schiaffino et al., 2013; Sousa-Victor et al., 2022). Myogenesis dysregulation causes muscle wasting diseases such as sarcopenia and cachexia, increasing the risk of frailty, morbidity, and lethality (Nishikawa et al., 2021a; Wiedmer et al., 2021). Numerous studies have discovered that muscle wasting is caused by various factors that inhibit myogenesis, such as oxidative stress, mitochondrial malfunction, and aging (Bonaldo and Sandri, 2013; McCarthy and Berg, 2021). Obesity has been shown to aggravate the negative effects of muscle loss, leading to sarcopenic obesity (Roh and Choi, 2020; Nishikawa et al., 2021b). Additionally, obesity reduces the capacity of skeletal muscle differentiation and may impact muscle plasticity and function (Brown et al., 2015). Furthermore, another study showed that the low level of inflammation induced by obesity downregulates myogenesis (Du et al., 2010). A deep understanding of myogenesis is crucial for comprehending the mechanisms regulating skeletal muscle mass under pathological disorders. The myogenic differentiation process comprises multiple pathways, and numerous potential targets in myogenesis regulation remain unknown.

To discover the potential targets in the process of myogenic differentiation, we applied RNA sequencing to identify unknown genes that regulate myogenesis in a classical mouse cell model. As a result, we identified *apolipoprotein L9a (Apol9a)* as a key moderator regulating myogenic differentiation. *Apol9a* is a member of the murine apolipoprotein L gene family, and interferon-inducible mouse Apol9a is secreted by macrophages to promote epithelial cell proliferation (Kreit et al., 2015; Sun et al., 2015). However, the biological function of Apol9a in skeletal muscles remains unclear. Experiments have indicated that *Apol9a* knockdown impairs skeletal muscle differentiation, principally by activating the ERK1/2 pathway. Activated ERK1/2 signaling promotes skeletal muscle cell proliferation but negatively regulates myogenic differentiation (Jones et al., 2001) and modulates nuclear factor of activated T cells c1 (NFATc1) (Chen et al., 2017). Studies have indicated that ERK1/2 activation is elevated in atrophic and damaged skeletal muscles (Penna et al., 2010). Our results highlight the potential importance of *Apol9a* in myogenic differentiation, suggesting that modulation of *Apol9a*-ERK activity may help reduce the risk of obesity-related muscle atrophy.

## Materials and methods

### Animals

Six-week-old male C57BL/6 mice were purchased from Jicui Yaokang Biological Technology Co., (Nanjing, China). All mice were maintained under standard conditions of 22°C ± 2°C, 50%–60% relative humidity, and alternate dark/light cycles. Mice were fed a high-fat diet (HFD) (60 kcal%, D12492, Research Diets) for 10 weeks to induce obesity. Control mice were fed a basal diet (10 kcal%, D12450B, Research Diets). Both groups of mice were sacrificed at 16 weeks of age, and their gastrocnemius muscles were dissected for experimental analyses. Skeletal muscle triglyceride (TG) contents were measured using a Triglyceride Quantification Kit (Nanjing Jiancheng, China). All experiments in this study were authorized by the Ethics Committee of Jiangnan University [NO: JN. No20211030c0700625 (426)].

### Cell culture and treatment

C2C12 mouse myoblast cells were purchased from the ATCC (CRL-1772, United States). The C2C12 cell line was cultured in growth medium (GM) supplemented with basic DMEM (11995–065, Gibco, United States), 10% FBS (1009–141, Gibco, United States), and 1% penicillin plus 1% streptomycin (SV30010, Hyclone, United States) at 37°C and 5% CO_2_. GM was replaced with differentiation medium (DM) containing DMEM with 2% horse serum (HS, 16050–130, Gibco, United States) and incubated for 4 days in order to induce C2C12 cell differentiation when C2C12 cells reached 90% confluency.

To explore the effect of *Apol9a* on C2C12 cells, small interfering RNA (siRNA, GenePharma, Shanghai, China) was used to knockdown intracellular *Apol9a*, and the overexpression plasmid (GENEWIZ) was used to overexpress *Apol9a*. C2C12 cells were seeded into 6-well plates and cultured for 8 h (equivalent to a cell density of 30%–40%). Next, siRNA and the overexpression plasmid were transfected into cells at a concentration of 50 nM. The jetPRIME^®^ transfection reagent (114–15, Polyplus transfection) was used to transfer siRNAs, the overexpression plasmid, and the empty control plasmid. At 12 h after transfection, the transfection medium was replaced with DM to induce differentiation for 4 days. Specific knockdown of *Apol9a* was validated using two different siRNAs. The two siRNA sequences for mouse *Apol9a* was 5′-UUGUAUCCAAGGCCAAGUUGUTT-3′and 5′-AGC​CCU​UGA​GCA​GCA​CAU​GAA​TT-3′. A random siRNA sequence (A06001, GenePharma, shanghai, China) was applied as control. To inhibit the ERK1/2 signaling pathway, C2C12 myoblasts were treated with 50 μm PD98059 (HY-12028, Med Chem Express) in DM for 4 d (after 8 h transfection). Dimethyl sulfoxide (DMSO, D8418, Sigma-Aldrich) was used as a solvent control.

Myotube diameter measurements were obtained using ImageJ software. The diameter was measured at the widest region of each myotube, and the mean diameter size was compared between conditions.

### qPCR

Total RNA was isolated from both C2C12 myoblasts and skeletal muscle tissue by using the RNA extraction kit (K101, JN. BIOTOOLS, Wuxi, China). cDNA was synthesized using the BTS I 1st Strand cDNA Synthesis Kit (K102, JN. BIOTOOLS, Wuxi, China), and 1 μg of total RNA to be used for quantitative real-time PCR (qPCR) was reverse transcribed. qPCR was performed using the Power SYBR Green Master Mix kit (4367659, Invitrogen, United States) in a CFX 96^TM^RealTime PCR Detection System (Bio-Rad, United States). The amplification conditions for qPCR were as follows: 94°C for 5 min, followed by 45 cycles of 94°C for 15 s and 45 s at 59°C. The fold change of gene expression was analyzed according to the 2^−ΔΔCT^ method. *β-actin* was utilized as the internal control for normalization. The sequences of all primers used in this research are presented in Supplementary Table S1.

### Western blotting

Total proteins were extracted from the cell lysate and determined using the Quick BCA Protein Assay Kit (SYW3-1, Solarbio, China). The lysate proteins were resolved through SDS-PAGE with a 10% gel, transferred to PVDF membranes (IPVH00005, Merck Millipore), blocked, and incubated with primary antibodies against Apol9a (AC-15–1076, Ango Biotechnology, 1:1000); myosin heavy chain (MyHC) (MF-20, 1:1000, Developmental Studies Hybridoma Bank); myogenin (MyoG) (ab 1835, Abcam, 1:2000); myoblast determination protein (MyoD) (18943-1-AP, 1:1000, Proteintech); Myf5 (BD-PT2930, 1:1000, Biodragon); phospho-Erk1/2 (4370, 1:1000, CST); Erk1/2 (4695, 1:1000, CST); phospho-JNK (4668, 1:1000, CST); JNK (9252, 1:1000, CST); phospho-p38 (4511, 1:1000, CST); p38 (9212, 1:1000, CST); and β-actin (ab8227, 1:2000, Abcam). After all membranes were washed, they were treated with specific secondary antibodies (AS014, AS003, Abclonal, China). Specific protein bands were detected using an ECL kit (WBKLS0100, Merck Millipore). The images were observed using the Bio-Rad ChemiDoc MP Imaging System, and then, image bands were quantified through densitometry by using ImageJ plus software. The expression level of each target protein was normalized to that of β-actin, which was used as an internal control.

### Immunocytochemistry

C2C12 myoblasts were fixed with paraformaldehyde (4%) for 20 min and treated with 1% Triton X-100 for 5 min. The fixed cell samples were incubated with blocking buffer (A8010, Solarbio, China) in phosphate-buffered saline (PBS) and then incubated with anti-MyHC (MF20, 1:50, DSHB) antibody at 4°C overnight. After washing the samples with PBS, the cell samples were treated with a 1:100 dilution of secondary antibody, which was derived from goat anti-mouse IgG (AS001, Abclonal) and conjugated with FITC, for 2 h. Cell nuclei were counterstained with DAPI staining solution (C1002, Beyotime, China). Finally, the stained cells were viewed using a fluorescence microscope (Leica DM2500, Leica Microsystems), and the images were captured. The fusion index was calculated as the percentage of nuclei in fused myotubes out of the total nuclei. The number of nuclei in each image was evaluated using the ImageJ plus software.

### Fatty acid methyl ester analysis

Fatty acid extraction and methyl-esterification were performed as previously described (Zhu et al., 2022a). Briefly, the recovered fatty acid methyl esters were examined through GC-MS (QP2010 ultra mass spectrometer, GC 2010 plus, Thermo Scientific). The electron energy was fixed at 70 eV, and the temperature programming profile was as follows: kept for 5 min at 60°C, up to 120°C in increments of 10°C/min, maintained for 5 min at 120°C, up to 190°C in increments of 5°C/min, kept for 7 min at 190°C, up to 230°C in increments of 2°C/min, and kept for 10 min at 230°C. A 5-MS column (Restek, United States) was used to resolve all samples. The detector and ion source were run at 240°C and 230°C, respectively. The peaks obtained were identified by comparing their retention times with those of known standards (Sigma). Pentadecanoic acid (C15:0) was used as the internal standard, and each sample was normalized to total cellular protein concentration, as measured using the Quick BCA protein assay kit (SYW3-1, Solarbio, China).

### Lipidomic analysis

Cell samples were produced in the manner previously described (Zhu et al., 2022b). Lipidomic analysis was conducted using LC-MS (QExactive Plus Orbitrap mass spectrometer, Thermo Scientific). Acetonitrile:MilliQ water (6:4 v/v) and isopropanol:acetonitrile (9:1 v/v) were used as solvents A and B, respectively; both solvents contained 10 mm ammonium acetate. Column chromatography was performed using the Waters ACQUITY UPLC CSHTM C18 column. The gradient profile was as follows: 32%–100% solvent B over 24 min, back to 32% solvent B and 6 min before the next injection, and equilibrate the column. LipidSearch program v4.1.16 (Thermo Scientific) was used to identify the type of lipids. A pool of all lipid extracts was prepared and used for quality control. SIMCA-P (Sweden) was used to import the raw MS data, and an orthogonal projection was performed for latent structures-discriminant analysis (OPLS-DA). The resulting heatmap plots were drawn using R software.

### RNA sequencing

RNA sequencing (RNA-seq) was conducted as previously described (Zhang et al., 2021; Zhu et al., 2022c). Briefly, total RNA of C2C12 myoblasts transfected with si-NC or si-*Apol9a* was isolated using the RNA extraction kit (K101, JN. BIOTOOLS, China). Library construction and paired-end sequencing were performed by GENEWIZ Biotech (Suzhou, China). Raw data files were matched to the mouse reference genome by using STAR software (http://www.code.google.com/p/rna-star/). For each sample, fold change was estimated using the fragments per kilobase per million reads values, and differential expression analysis was performed using the DESeq2 package. The standard of fold change ≥1.5 and *p* < 0.05 were defined to screen differentially expressed genes (DEGs). Gene ontology (GO) analysis was conducted to perform the functional enrichment analysis of specific DEGs using Metascape (metascape.org). All RNA-seq data files have been deposited in the SRA database (Accession: PRJNA845924).

### Statistical analysis

Statistical analysis was performed using SPSS software version 22.0 and GraphPad Prism 8.0. The results are shown as mean ± SEM from at least three independent experiments. Differences between groups were evaluated using an unpaired Student’s t-test (between two groups) or one-way ANOVA (between multiple groups). *p < 0.05, **p < 0.01, and ***p < 0.001 were considered statistically significant.

## Results

### Skeletal muscle mass and differentiation ability decrease in obese mice

**FIGURE 1:**
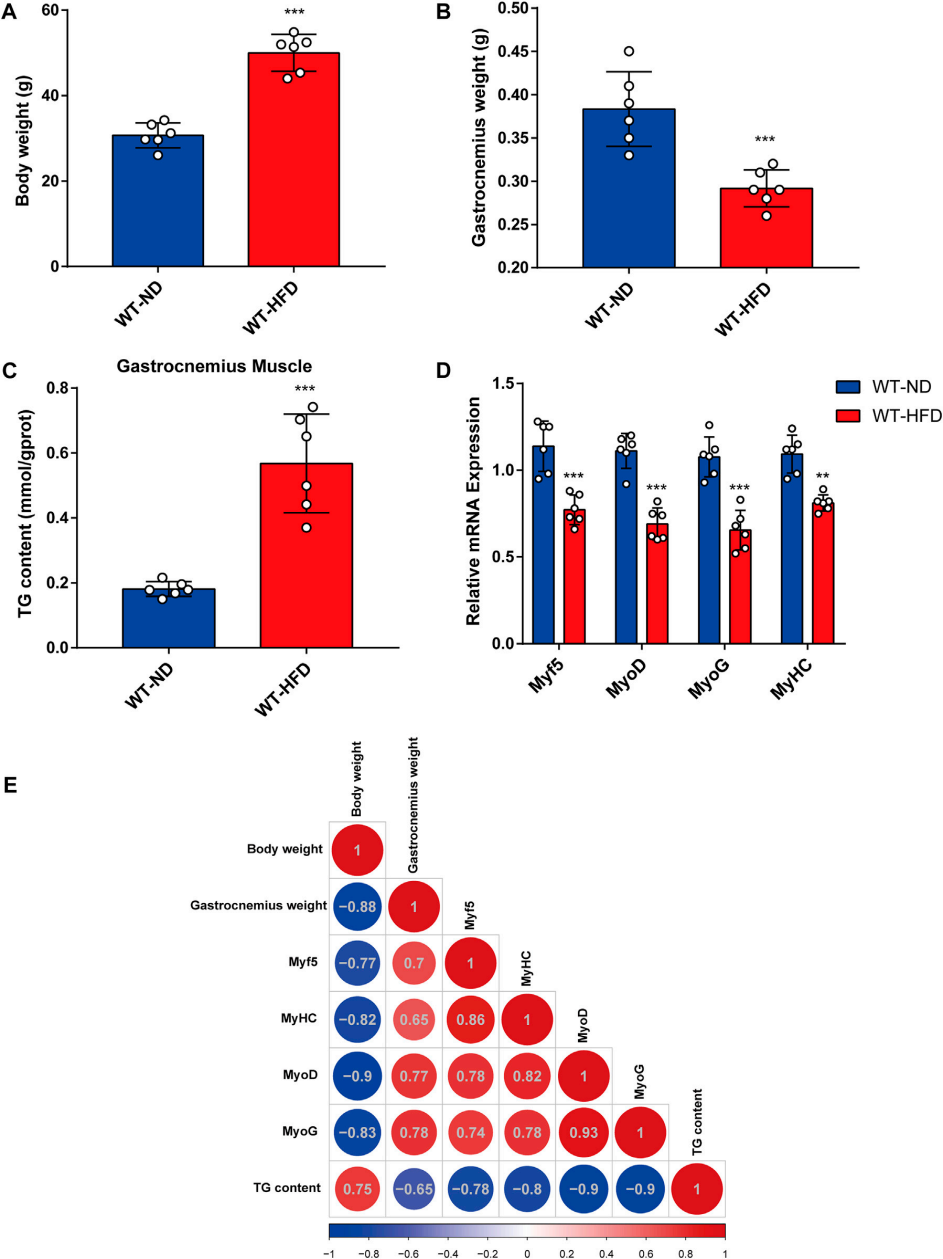
Correlation between obesity and myogenic differentiation. Six-week-old male mice were divided into two groups, each fed with normal chow and high-fat diet for 8 weeks. **(A)** Body weight comparison. **(B)** Gastrocnemius muscle weight (GW) comparison. **(C)** Comparison of triglyceride (TG) levels in gastrocnemius muscles from the ND-fed and HFD-fed mice as assessed using the Triglyceride Quantification Kit. **(D)** The mRNA expression levels of myogenesis-related genes (*Myf5*, *MyoD*, *MyoG*, and *MyHC*) in the ND-fed and HFD-fed mice as measured through quantitative real-time PCR (qPCR). **(E)** Correlation heatmap (Pearson correlation) of body weight, TG level, GW, and mRNA levels of myogenesis-related genes. Positive and negative correlations are shown by red and blue colors, respectively. Date are shown as mean ± SEM (*n* = 6 for each group). *p < 0.05, **p < 0.01, and ***p < 0.001 vs. WT-ND.

To evaluate the effects of obesity on skeletal muscle differentiation, we used the HFD-induced obesity mouse model (Feraco et al., 2021). Body weight and TG content in skeletal muscle tissue were considerably higher in HFD mice than in normal diet (ND) mice (Figures 1A,C). Meanwhile, the HFD mice exhibited a lower gastrocnemius muscle mass (Figure 1B) than the ND mice. Furthermore, a significant decrease in the mRNA expression levels of myogenesis-related genes (including *Myf5*, *MyoD, MyoG*, and *MyHC*) was observed in the gastrocnemius muscle from HFD mice (Figure 1D). Based on Pearson’s correlation analysis, we investigated the correlations between body weight, gastrocnemius muscle mass, expression levels of myogenesis-related genes, and TG levels in HFD mice (Figure 1E). A notable negative correlation was observed between TG content and gastrocnemius muscle mass. Together, these results demonstrate that muscle mass and myogenesis capacity were decreased in obese mice.

### *Apol9a* levels increase during myogenic differentiation and decrease in obese mice

**FIGURE 2:**
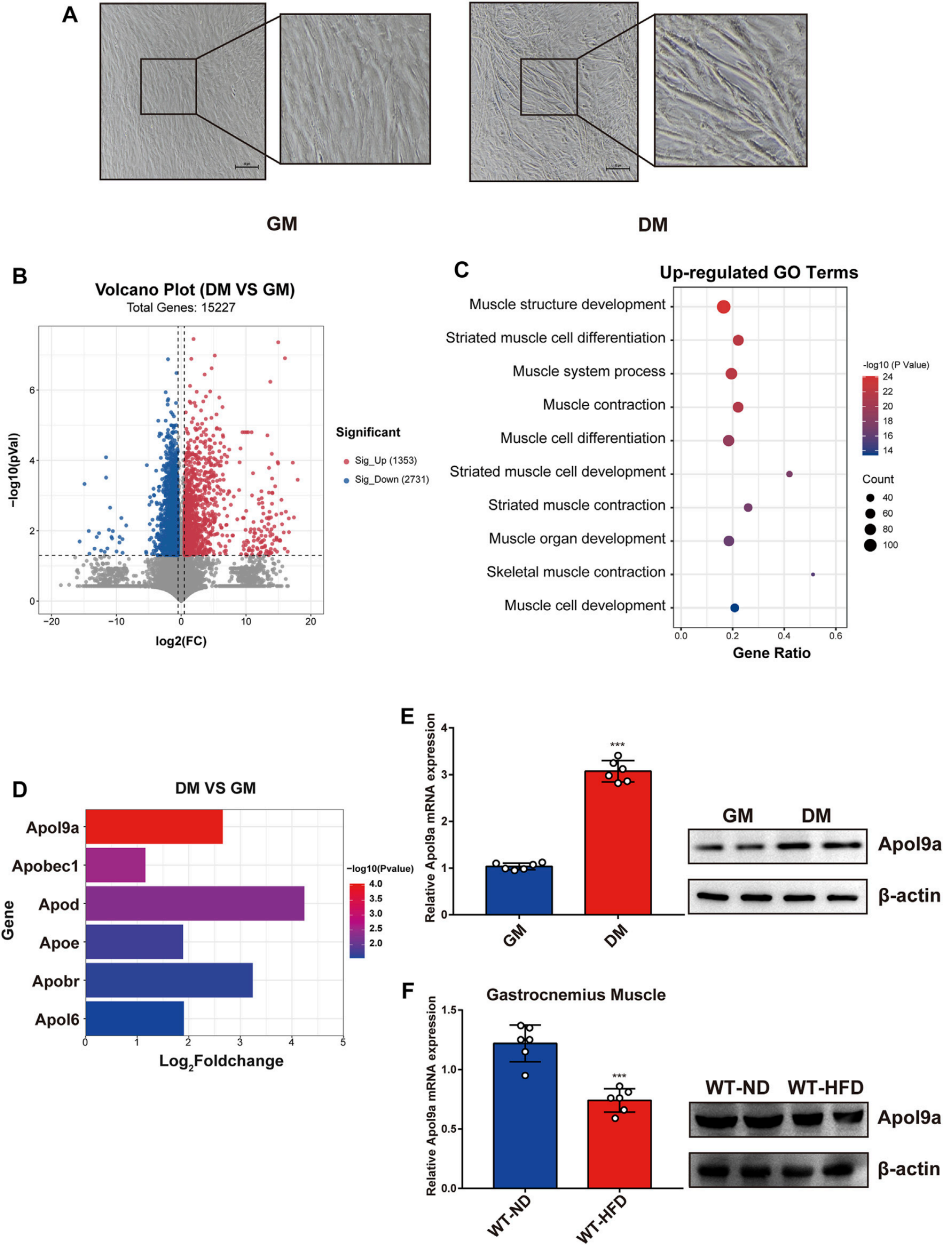
*Apol9a* is increased in myogenic differentiation and decreased in obese mice. RNA sequencing (RNA-seq) data of C2C12 cells with or without myogenic induction were collected. **(A)** Representative images from C2C12 cells in growth medium (GM) and differentiation medium (DM). Scale bar, 100 μm. **(B)** Volcano plot showing differentially expressed genes (DEGs) in differentiated or undifferentiated C2C12 cells. **(C)** Ten most upregulated GO terms during C2C12 differentiation according to the RNA-seq data. **(D)** Expression profiles of apolipoprotein family genes during C2C12 differentiation. **(E)** The relative mRNA expression (left) and protein levels (right) of *Apol9a* during C2C12 differentiation were measured using qPCR and western blotting. **(F)** The relative mRNA expression (left) and protein levels (right) of *Apol9a* in obese skeletal muscle were measured through qPCR and western blotting. The data are presented as mean ± SEM from at least three separate experiments. *p < 0.05, **p < 0.01, ***p < 0.001.

To determine the critical genes involved in myogenic differentiation, we compared the transcriptome of differentiated C2C12 cells (an established myoblast cell model) with that of undifferentiated C2C12 cells (Yaffe and Saxel, 1977). After myogenic differentiation, C2C12 cells became fused and the myotube diameter increased (Figure 2A). As demonstrated in the volcano plot (Figure 2B), 1353 genes were upregulated following myogenic differentiation and 2731 genes were downregulated (fold change ≥1.5). The upregulated genes were subjected to GO analysis, and the top 10 enriched GO terms were all related to muscle differentiation (Figure 2C). Among the upregulated gene sets, we observed that many apolipoprotein family genes were significantly upregulated following myogenic differentiation (Figure 2D). The most significant change involved *Apol9a*, an understudied cytoplasmic, interferon-inducible gene with antiviral activity (Kreit et al., 2015). To our knowledge, a role for *Apol9a* in myogenic differentiation has not been previously reported. Using qPCR, we confirmed that *Apol9a* mRNA expression levels increased following myogenesis (Figure 2E). However, *Apol9a* mRNA expression levels decreased in obese mice (Figure 2F). Together, these results indicate that *Apol9a* may play a role in myogenic differentiation.

### Silencing *Apol9a* inhibits myogenic differentiation and *Apol9a* overexpression promotes myogenic differentiation

**FIGURE 3:**
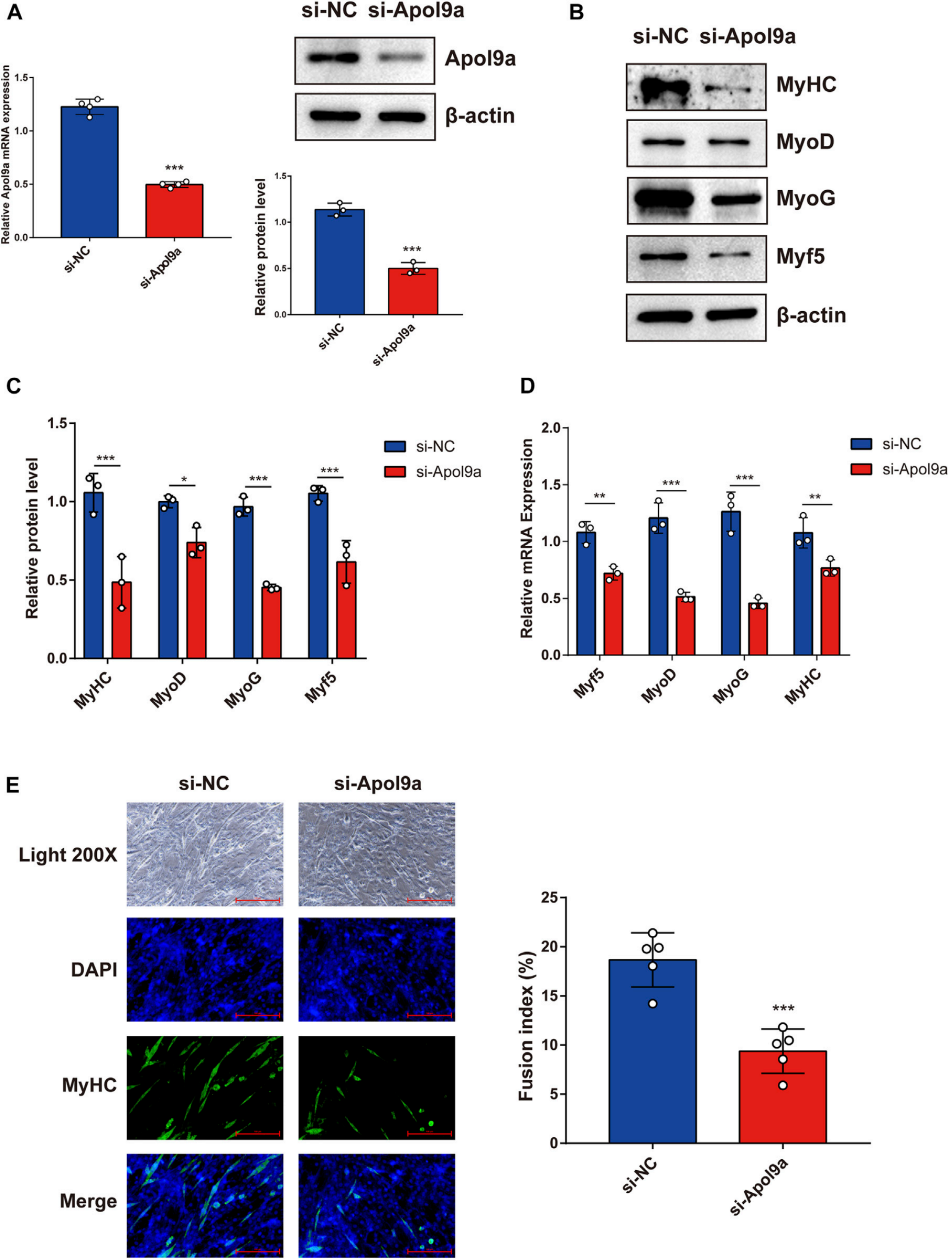
*Apol9a* knockdown inhibits C2C12 myogenesis. C2C12 cells were induced to differentiate after si-NC or si-*Apol9a* transfection for 4 days. **(A)** qPCR and western blot validation of the efficiency of *Apol9a* knockdown in C2C12 cells. **(B)** Protein expression levels of MyHC, MyoD, Myf5, and MyoG were estimated through western blot analysis after si-NC and si-*Apol9a* transfection. **(C)** Gray scale analysis of western blot results of **(B)** determined using ImageJ software. **(D)** qPCR was performed to assess the mRNA expression levels of several myogenic differentiation genes (*Myf5*, *MyoD*, *MyoG*, and *MyHC*). **(E)** Myotube formation can be observed in fluorescence images of DAPI (blue) and MyHC antibody-stained (green) myotubes of C2C12 cells transfected si-NC or si-*Apol9a*. Scale bar, 100 μm. The fusion index (%) was quantified and is shown on the right. The data are expressed as mean ± SEM of at least three independent experiments. *p < 0.05, **p < 0.01, ***p < 0.001 vs. si-NC control.

**FIGURE 4:**
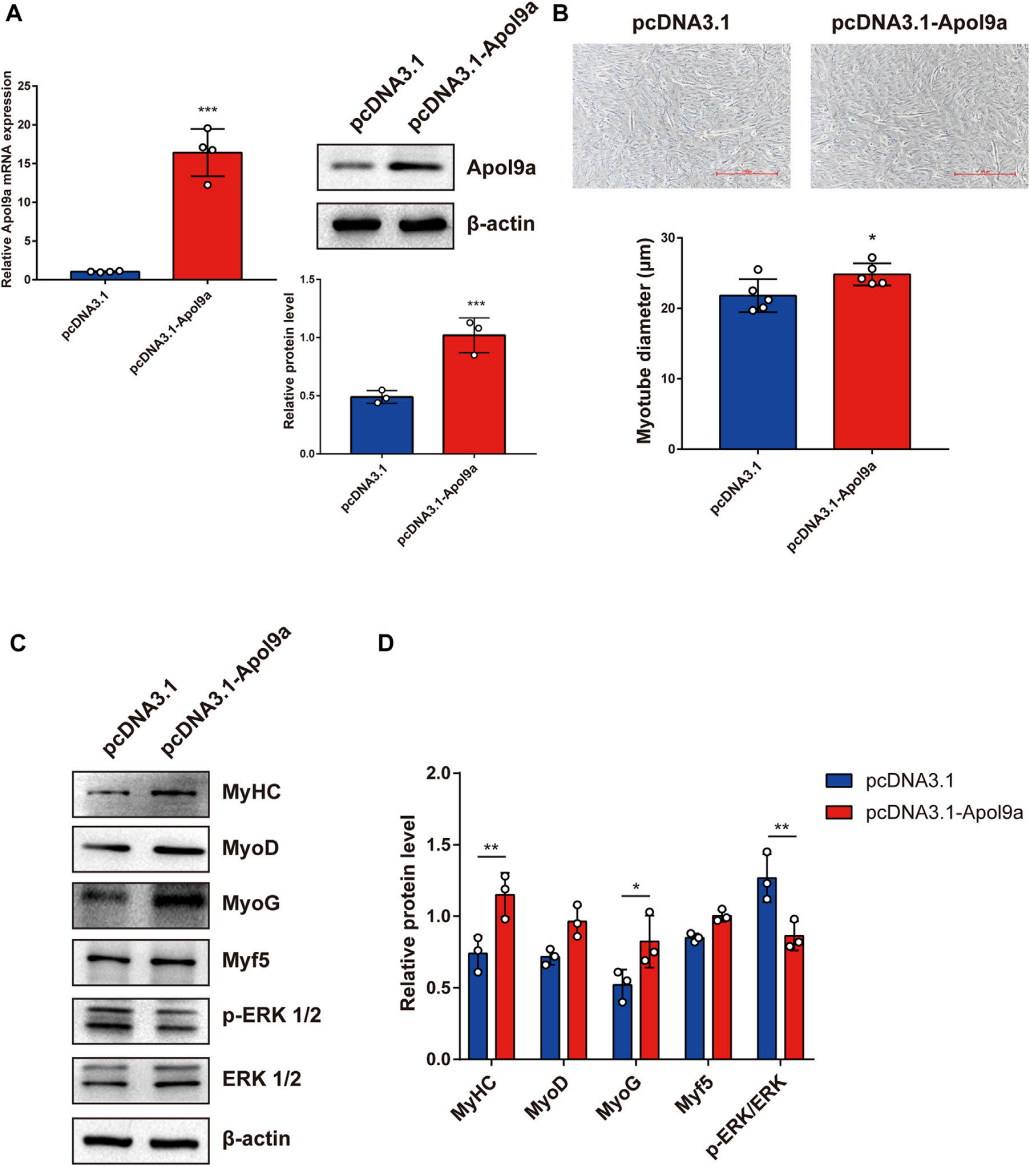
*Apol9a* overexpression promotes myogenic differentiation. C2C12 cells were induced to differentiate after pcDNA3.1 or pcDNA3.1-*Apol9a* transfection for 4 days. **(A)** qPCR and western blot validation of the efficiency of *Apol9a* overexpression in C2C12 cells. **(B)** Morphology of C2C12 cells treated with pcDNA3.1 or pcDNA3.1-*Apol9a*. The results of quantification of average myotube diameter are shown in the right. **(C)** Protein expression levels of MyHC, MyoD, Myf5, MyoG, p-ERK 1/2, ERK 1/2, and β-actin were estimated through western blot analysis after transfection. **(D)** Gray scale analysis of western blot results of **(C)** determined using ImageJ software. The data are expressed as mean ± SEM from at least three independent experiments. *p < 0.05, **p < 0.01, ***p < 0.001 vs. pcDNA3.1 group.

To further investigate the function of *Apol9a* in the progress of myogenesis, siRNA was used to interfere with *Apol9a* expression. C2C12 cells were transfected with *Apol9a* siRNA or NC siRNA, and then, the GM was replaced with DM for 4 days. *Apol9a* mRNA expression levels were reduced approximately 60% in *Apol9a* siRNA-transfected cells compared with the si-NC control group (Figure 3A). Myogenic differentiation is known to start with the induction of a specific set of transcription genes known as myogenic regulatory factors (MRFs), which include *Myf5*, *MyoD*, and *MyoG* (Chargé and Rudnicki, 2004; Ciciliot and Schiaffino, 2010). Comparison with the si-NC control group, the protein and mRNA expression levels of several myogenic differentiation markers, including Myf5, MyoD, MyoG, and MyHC, were remarkably decreased in differentiated C2C12 cells after si-*Apol9a* transfection (Figures 3B–D). To validate the effect of *Apol9a* deficiency on myotube formation, we evaluated the expression of MyHC protein through immunofluorescence in differentiated C2C12 cells and a quantitative analysis of the fusion index. As shown in Figure 3E, *Apol9a* knockdown cells displayed a different morphology and appeared to have shorter myofibers compared with the control group. Moreover, the fusion index was significantly decreased after *Apol9a* silencing (Figure 3E). Furthermore, we explored the effect of *Apol9a* overexpression on myogenic differentiation. Compared with the empty vector group, the Apol9a mRNA expression level was markedly increased in pcDNA3.1-*Apol9a*-transfected cells. Compared with the empty vector group, the Apol9a mRNA expression level was significantly increased in pcDNA3.1-*Apol9a*-transfected cells (Figure 4A), and the myotube diameter was significantly longer after *Apol9a* overexpression (Figure 4B). Western blot analysis showed that *Apol9a* overexpression significantly upregulated MyoG and MyHC protein levels (Figures 4C,D). Together, these results demonstrate that *Apol9a* knockdown inhibited myogenesis and *Apol9a* overexpression promoted myogenic differentiation.

### *Apol9a* silencing decreases the content of cellular fatty acids

**FIGURE 5:**
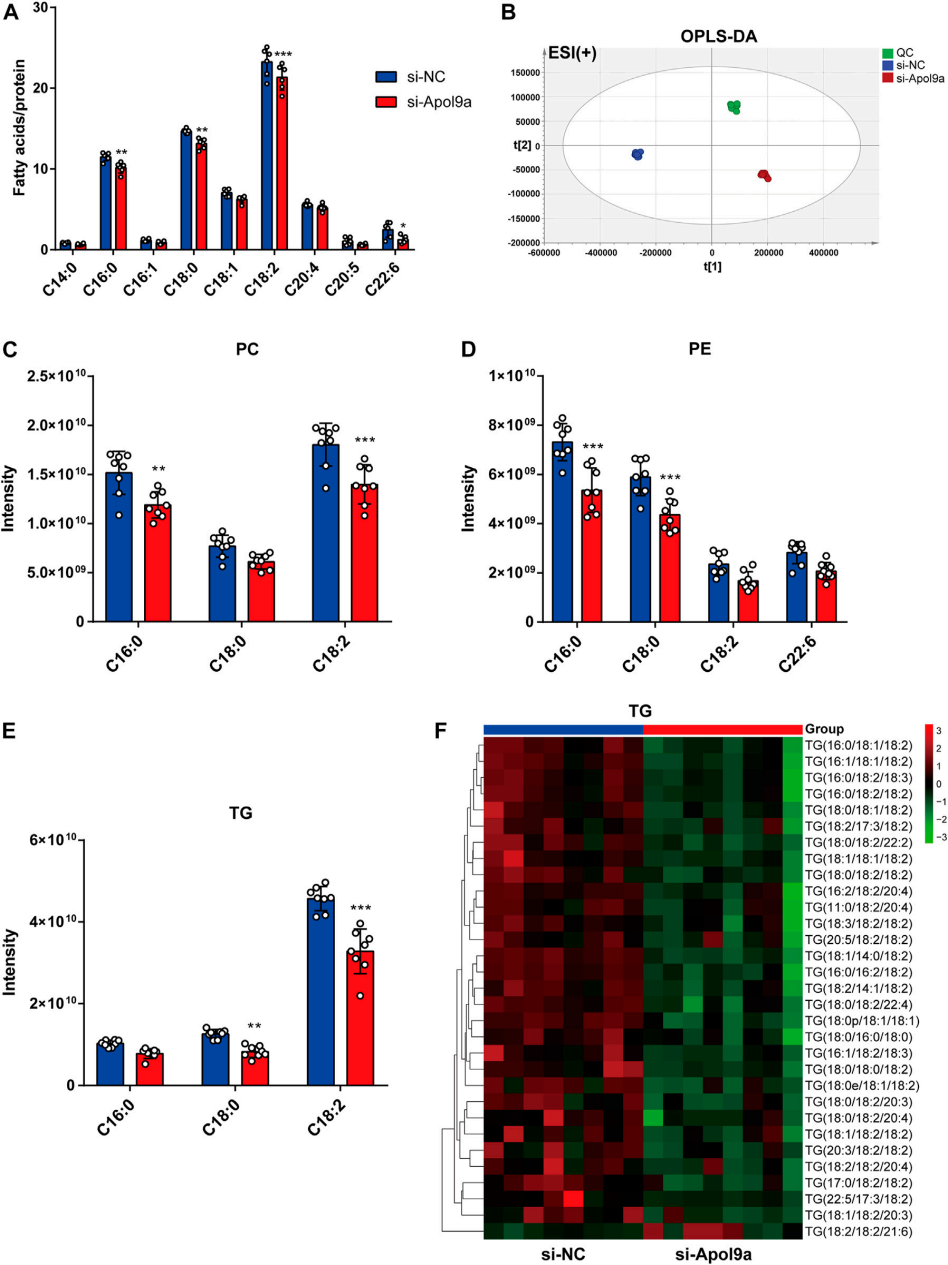
Fatty acid methyl ester analysis and lipidomic analysis. After myogenic differentiation for 4 days, C2C12 cells transfected with si-NC or si-*Apol9a* were collected, and cellular fatty acid and lipidomic analyses were performed. **(A)** Cellular fatty acid profiles were detected through GC-MS following C2C12 differentiation. **(B)** orthogonal partial least squares discriminant analysis (OPLS-DA) of lipid profiles in positive ion modes from C2C12 cells with or without *Apol9a* knockdown. **(C)** The composition of phosphatidylcholine (PC) (C16:0, C18:0, C18:2) among all lipids. **(D)** The composition of phosphatidylethanolamine (PE) (C16:0, C18:0, C18:2, C22:6) among all lipids. **(E)** The composition of triacylglycerol (TG) (C16:0, C18:0, C18:2) among all lipids. **(F)** Heatmap showing the differential lipid composition of TGs (C18:0, C18:2). The data are presented as mean ± SEM (*n* = 8, each group). *p < 0.05, **p < 0.01, ***p < 0.001 vs. si-NC group.

*Apol9a* is an apolipoprotein family member, and previous research has suggested that *Apol9a* has a role in lipid transport (Arvind and Rangarajan, 2016). To identify changes in the types of fatty acids and changes in lipid composition induced by *Apol9a* knockdown after cell differentiation, the types and contents of fatty acid in C2C12 cells with or without *Apol9a* knockdown after myogenic differentiation were determined through GC-MS and LC-MS, respectively. After *Apol9a* knockdown, the levels of various fatty acids (C16:0, C18:0, C18:2, and C22:6) significantly decreased (in comparison with the si-NC transfected cells) (Figure 5A). To further analyze changes in lipid composition, we applied LC-MS-based lipidomics to identify the specific types of lipids whose levels decreased in *Apol9a* knockdown cells. Moreover, orthogonal partial least squares-discriminant analysis (OPLS-DA) indicated different clustering of lipids from NC and *Apol9a*-silenced groups (Figure 5B). While the intensity levels of C16:0 fatty acid were mainly decreased in phosphatidylcholine (PC) and phosphatidylethanolamine (PE), those of C18:0 fatty acid were reduced in TG and PE (Figure 5D). In addition, a significant decrease was observed in the intensity levels of C18:2 fatty acid, especially in PC and TG (Figures 5C,E). Elevated skeletal muscle TG is reported to be related to insulin resistance in obesity and impaired skeletal muscle differentiation (Wu and Ballantyne, 2017), (Eum et al., 2020). Furthermore, our heatmap analysis revealed that many TG species (including C18:0 and C18:2 fatty acids) demonstrate a remarkable decrease (VIP >1 and *p* < 0.05) in C2C12 cells after *Apol9a* knockdown and myogenic differentiation (Figure 5F). Together, these results provide evidence that inhibition of myogenesis following *Apol9a* knockdown may be related to changes in lipid composition, especially changes in TG species.

### RNA-seq analysis reveals the ERK1/2 pathway as a downstream target of *Apol9a*

**FIGURE 6:**
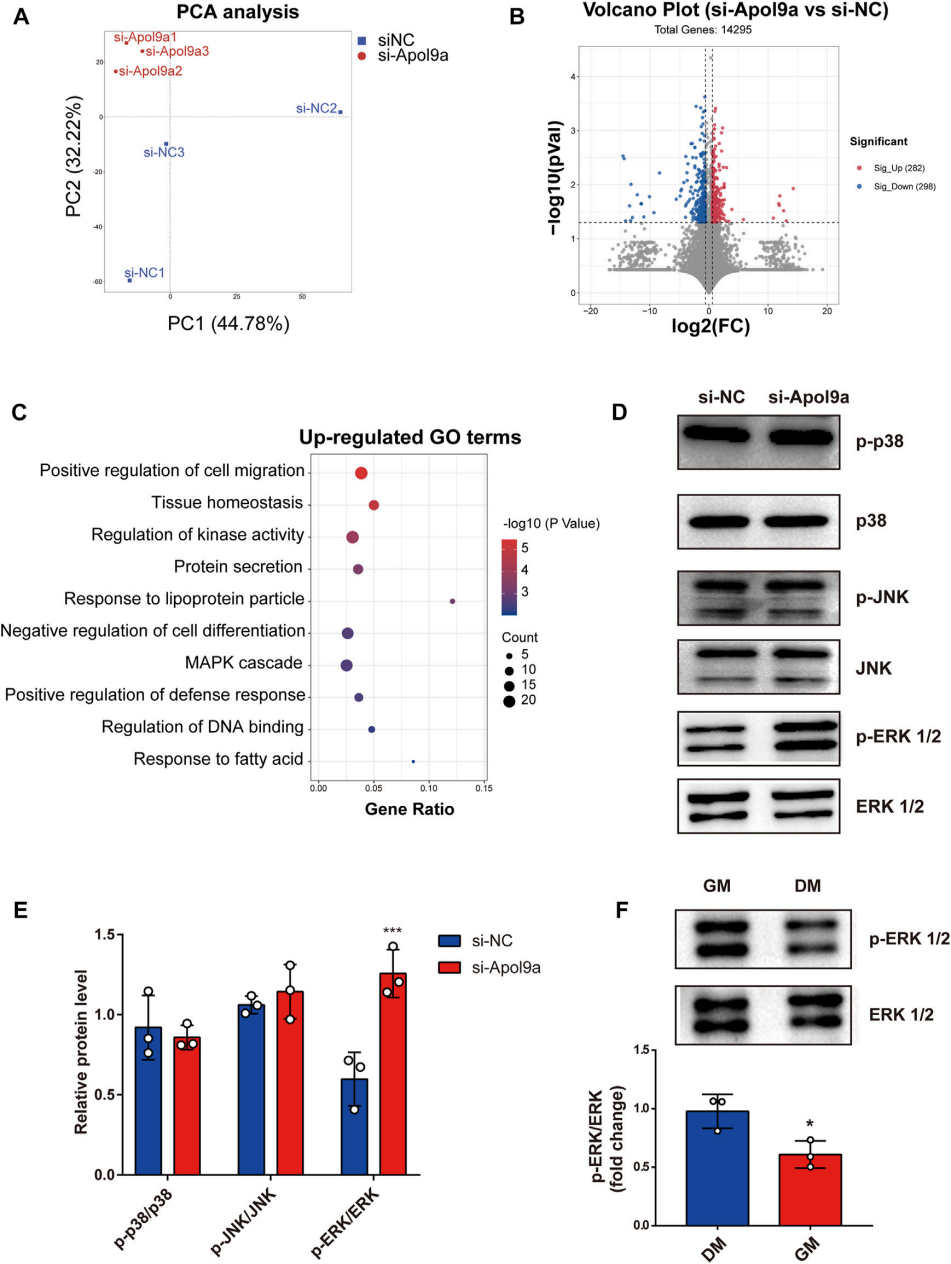
Transcriptomic data. C2C12 cells with or without si-*Apol9a* treatment was harvested after myogenic differentiation, and then transcriptomic analysis was performed. **(A)** Principal component analysis of RNA-seq data from C2C12 cells with (si-*Apol9a*) or without (si-NC) *Apol9a* knockdown after myogenic differentiation (*n* = 3 per group). **(B)** Volcano plot showing the DEGs in C2C12 cells with (si-*Apol9a*) or without (si-NC) *Apol9a* knockdown after myogenic differentiation. **(C)** Gene ontology analysis of upregulated DEGs. **(D)** Western blot analysis showed the protein levels of p-P38, P38, p-JNK, JNK, p-ERK1/2, and ERK1/2. **(E)** Gray scale analysis of western blot results determined using ImageJ software. **(F)** Western blot analysis for p-ERK1/2 and ERK1/2. C2C12 cells were cultured in GM or DM for 4 days. The data are expressed as mean ± SEM. *p < 0.05, **p < 0.01, ***p < 0.001 vs. si-NC group.

To elucidate the underlying molecular mechanisms of *Apol9a* regulation of myogenesis, RNA sequencing was used to evaluate gene expression differences in C2C12 cells with or without *Apol9a* knockdown. Principal component analysis (PCA) was then performed to discriminate changes in expression between experimental groups. The PCA results revealed that the two groups could be completely separated based on their transcriptomes (Figure 6A). Based on the criteria of fold change ≥1.5 and *p* < 0.05, 580 differentially expressed genes (DEGs) between the two groups were identified, including 282 downregulated genes and 298 upregulated genes (Figure 6B). Then, GO analysis was applied to find the potential functions of the upregulated and downregulated genes. As shown in Figure 6C, significantly enriched functional terms, including “tissue homeostasis,” “response to lipoprotein particle,” “negative regulation of cell differentiation,” and “MAPK cascade,” were identified using the upregulated gene set. However, no specifically enriched biological processes were identified using the downregulated gene set (data not shown). The MAPK cascade pathway is essential for the regulation of myogenic differentiation (Jones et al., 2001; Keren et al., 2006; Xie et al., 2018; Boyer et al., 2019). We hypothesized that the ERK1/2 signaling pathway may play a role in the inhibition of myogenic differentiation induced by *Apol9a* knockdown. This hypothesis was subsequently confirmed by western blotting analyses of the ERK1/2 phosphorylation status and protein levels in C2C12 cells. Thus, while *Apol9a* knockdown markedly increased ERK1/2 phosphorylation during myogenesis, other MAPKs (JNK and p38) remained unchanged (Figure 6D). Moreover, phosphorylated ERK1/2 protein levels were significantly reduced after C2C12 differentiation (Figure 6E). In summary, our data demonstrate that *Apol9a* knockdown activates the ERK1/2 signaling pathway during myogenic differentiation.

### Inhibition of myogenic differentiation by Apol9a knockdown is reversed by an ERK inhibitor

**FIGURE 7:**
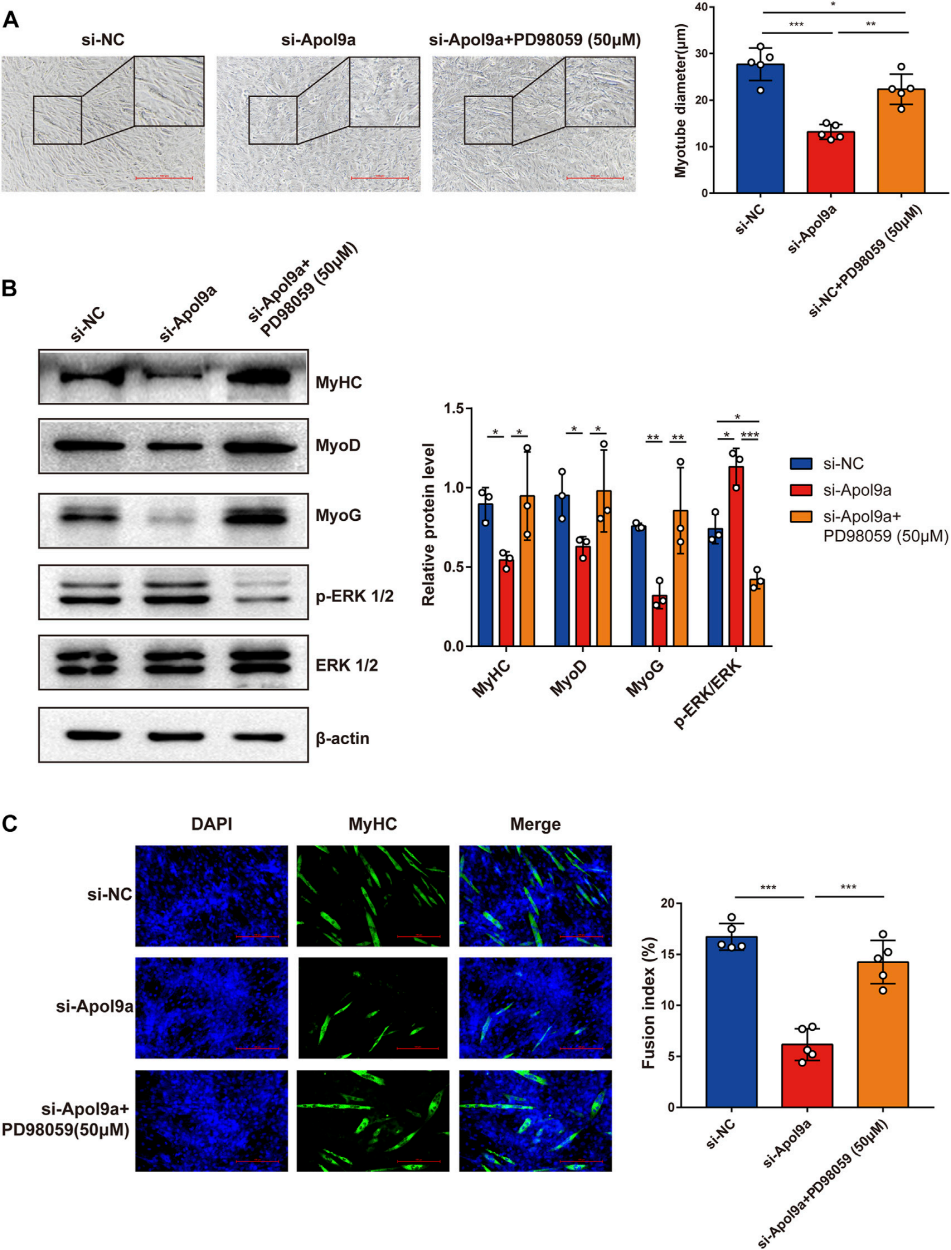
ERK inhibitor reverses *Apol9a* knockdown-induced inhibition of myogenesis. C2C12 cells were transfected with NC or *Apol9a* siRNA. After 12 h, the GM was replaced with the DM to induce differentiation or DM containing PD98059 (50 μm). **(A)** Morphology of C2C12 cells treated with si-NC, si-*Apol9a*, and si-*Apol9a* plus PD98059 (50 μm). The results of quantification of average myotube diameter are shown in the right. **(B)** Protein expression levels of MyHC, MyoD, MyoG, p-ERK1/2, and ERK1/2 as detected through western blot analysis. Gray intensity analysis is shown on the right. **(C)** Representative images of immunofluorescence for MyHC (green) are shown. The fusion index (%) was quantified and is shown on the right. Scale bar, 100 μm. Each experiment in this study was repeated at least three independent times. *p < 0.05, **p < 0.01, ***p < 0.001.

To test the hypothesis that *Apol9a* regulates myogenic differentiation *via* the ERK1/2 signaling-dependent pathway, we applied PD98059, a specific inhibitor of ERK1/2, to the C2C12 cells. In agreement with our hypothesis, *Apol9a* knockdown-induced inhibition of myogenesis was almost completely reversed by PD98059 (Figure 7A). Furthermore, western blot analysis and immunofluorescence experiments demonstrated that ERK inhibition blocked the downregulation of myogenic genes (*MyHC*, *MyoD*, and *MyoG*) otherwise observed during *Apol9a* knockdown (Figures 7B,C). Together, these results provide evidence that *Apol9a* regulates myogenic differentiation *via* the ERK pathway.

## Discussion

Skeletal muscle health is very crucial for human life at all stages. Moreover, skeletal muscle mass and myogenesis capacity are impaired in many pathological conditions, including obesity (Fu et al., 2016; Geiger et al., 2020). We confirmed that skeletal muscle mass and myogenesis capacity are decreased in obesity condition through the HFD-induced obesity mouse model. Although considerable attention has been paid to the investigation of therapeutic pharmacological supplements for improving muscle mass and function, progress is limited. Skeletal muscle plasticity is compromised due to impaired myogenesis (Suetta, 2017). By furthering our understanding of the molecular events regulating myogenesis, we may be able to develop an effective strategy for maintaining skeletal muscle integrity and plasticity.

In this study, *Apol9a* was identified as a new target gene critically involved in myogenesis. To our knowledge, this is the first study to report the underlying mechanisms and importance of *Apol9a* in skeletal muscle function. *Apol9a* belongs to the murine apolipoprotein L family and was previously reported to be an interferon-stimulated protein (Smith and Malik, 2009; Kreit et al., 2014). However, not much more is known about the function of *Apol9a*. In general, apolipoproteins play a role in lipid transportation (Su and Peng, 2020). For example, mouse apolipoprotein L9 is reported to be a PE-binding protein (Thekkinghat et al., 2019). In agreement with its proposed role in lipid transportation, our lipidomic data indicates that *Apol9a* knockdown decreased the PE level. Moreover, the TG and PC contents were reduced after *Apol9a* knockdown. Obesity leads to ectopic lipid deposition and abnormal lipid metabolism in skeletal muscle (Girousse et al., 2019). Our study confirmed that *Apol9a* is involved in multiple lipid transport activities, and its related functions should be further studied.

To explore the molecular mechanisms of *Apol9a* regulation of myogenic differentiation, RNA-seq was used to reconstruct *de novo* transcriptomes of *Apol9a* silenced cells. The results provide evidence that ERK signaling plays a crucial role in myogenesis. ERK1/2 signaling pathways are known to regulate numerous cellular processes, including cell proliferation, differentiation, and apoptosis (Cagnol and Chambard, 2010). While activated, ERK1/2 signaling plays a positive role in skeletal muscle cell proliferation, it negatively regulates myogenic differentiation (Jones et al., 2001). Moreover, studies have reported that ERK1/2 activation is elevated in atrophied and damaged skeletal muscles (Weston et al., 2003; Barreto et al., 2016; Oishi et al., 2019). Skeletal muscle differentiation is known to be mediated by several muscle regulatory factors, including MyoD, MyoG, and MyHC (Chal and Pourquié, 2017; Xu et al., 2017; Engquist and Zammit, 2021). *Apol9a* silencing significantly increased ERK1/2 phosphorylation and markedly suppressed the expression of muscle regulatory marker genes, such as *Myf5*, *MyoD*, *MyoG*, and *MyHC*. The experiments of *Apol9a* overexpression proved that *Apol9a* had a promoting effect on myogenic differentiation and significantly inhibited the ERK 1/2 pathway. In addition, MyoG and MyHC are the most significantly altered proteins corresponding to *Apo9a* overexpression. Application of an ERK inhibitor (PD98059) significantly reversed the aforementioned effects, indicating that *Apol9a*-ERK may be an upstream regulator of myogenic differentiation. Together, our results provide evidence that *Apol9a*-ERK may be a key therapeutic target for the management of skeletal muscle integrity and plasticity.

## Conclusion

In summary, we indicate that *Apol9a* is a novel regulator of myogenic differentiation. *Apol9a* mRNA expression levels were significantly increased during myogenesis and decreased during obesity-induced muscle atrophy. *Apol9a* knockdown inhibited myogenic differentiation, possibly *via* the ERK1/2 signaling pathway. This study broadens our knowledge of the myogenic differentiation process and identifies a promising therapeutic target for intervention in obesity-related muscle atrophy.

## Data Availability

The datasets presented in this study can be found in online repositories. The names of the repository/repositories and accession number(s) can be found below: https://www.ncbi.nlm.nih.gov/bioproject/PRJNA845924.

## Abbreviations

Apol9a, apolipoprotein L 9a; MyHC, myosin heavy chain; MyoD, myoblast determination protein; MyoG, myogenin; HFD, high-fat diet; qPCR, Real-time quantitative polymerase chain reaction; TG, triacylglycerol; PCA, principal component analysis; FAME, fatty acid methyl ester; OPLS-DA, orthogonal partial least squares-discriminant analysis; PE, phosphatidylethanolamine; PC, phosphatidylcholine; VIP, Variable Importance in Projection.
