# Supplementary material for: Apol9a regulates myogenic differentiation via the ERK1/2 pathway in C2C12 cells
Source: Front Pharmacol. 2022 Nov 23;13:942061. doi: 10.3389/fphar.2022.942061 (PMC9727217; doi:10.3389/fphar.2022.942061)
Supplement: Supplementary file 1 [file Table1.DOCX]

**Supplementary Table 1. Primer list (*β-actin* was utilized as the housekeeping gene)**

| **Gene** | **Primer pair** |
| --- | --- |
| *Apol9a* | (F) 5’-TCTGACATCCTGAGCCTCCTTGG -3’  (R) 5’-GCCAGTCGGAGCAGCTTCAAC -3’ |
| *MyHC* | (F) 5’-TTGAAAAGACGAAGCAGCGAC-3’  (R) 5’-AGAGAGCGGGACTCCTTCTG-3’ |
| *MyoG* | (F) 5’-GAGACATCCCCCTATTTCTACCA -3’  (R) 5’-GCTCAGTCCGCTCATAGCC -3’ |
| *Myf5* | (F) 5’-AAGGCTCCTGTATCCCCTCAC-3’  (R) 5’- TGACCTTCTTCAGGCGTCTAC-3’ |
| *MyoD* | (F) 5’-CCACTCCGGGACATAGACTTG -3’  (R) 5’-AAAAGCGCAGGTCTGGTGAG -3’ |
| *β-actin* | (F) 5'-GATCTGGCACCACACCTTCT-3'  (R) 5'-GG GGTGTTGAAGGTCTCAAA-3' |
