# Supplementary figures and images for: Apol9a regulates myogenic differentiation via the ERK1/2 pathway in C2C12 cells
Source: Front Pharmacol. 2022 Nov 23;13:942061. doi: 10.3389/fphar.2022.942061 (PMC9727217; doi:10.3389/fphar.2022.942061)

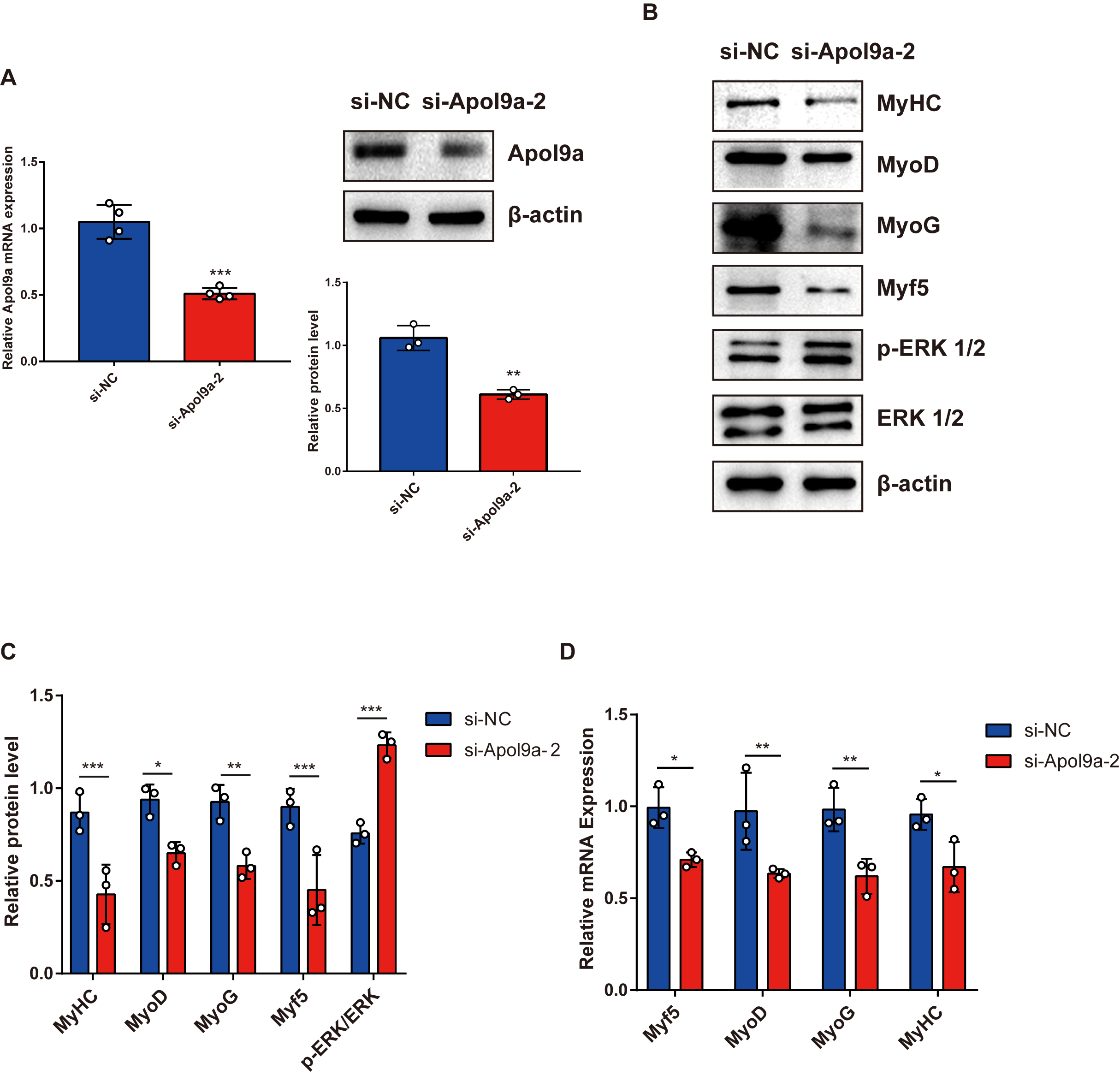

Supplement: Supplementary file 4 [file Image1.TIF]
